# Supplementary material for: The CD153 vaccine is a senotherapeutic option for preventing the accumulation of senescent T cells in mice
Source: Nat Commun. 2020 May 18;11:2482. doi: 10.1038/s41467-020-16347-w (PMC7235045; doi:10.1038/s41467-020-16347-w)
Supplement: Supplementary file 1 — Supplementary Information [file 41467_2020_16347_MOESM1_ESM.pdf]

Supplementary Information

The CD153 vaccine is a senotherapeutic option for preventing the accumulation of senescent T cells in mice

Shota Yoshida et al.

Supplementary Figure 1

A

Peptide #	Amino acid sequence
A	^{116} TKLSWNEDGT ^{125}
B	^{182} CESGVQSK ^{189}
C	^{101} KKSWAYLQ ^{108}
D	^{76} TTEKAPLKGG ^{85}
E	^{234} LYSSSD ^{239}

B

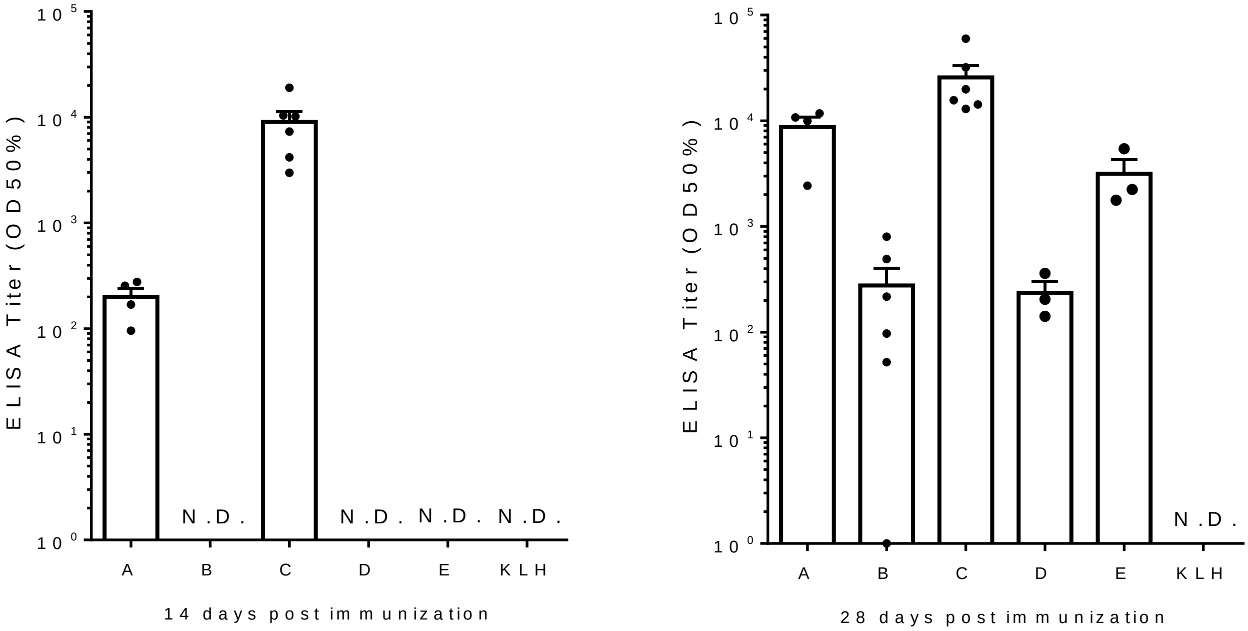

Seven-week-old C57BL/6J mice were immunized with the CD153-Alum vaccine (#A, #B, #C, #D and #E) or the KLH-Alum vaccine on days 0 and 14. The CD153-Alum vaccine was composed of 30 μg of the CD153 peptide (#A–#E) conjugated to KLH (200–300 μg) and mixed with Alum adjuvant, and the KLH-Alum vaccine was composed of KLH (200–300 μg) and mixed with Alum adjuvant. (**A**) Amino acid sequence of each peptide (#A–E). (**B**) The titer against CD153-BSA is expressed as the dilution of serum providing half-maximal binding (OD 50%) on days 14 and 28 postimmunization. #A, n = 4; #B, n = 6; #C, n = 6; #D, n = 3; #E, n = 3; KLH, n =6. All the data are expressed as the mean ± SEM; N.D., not detected.

Supplementary Figure 2

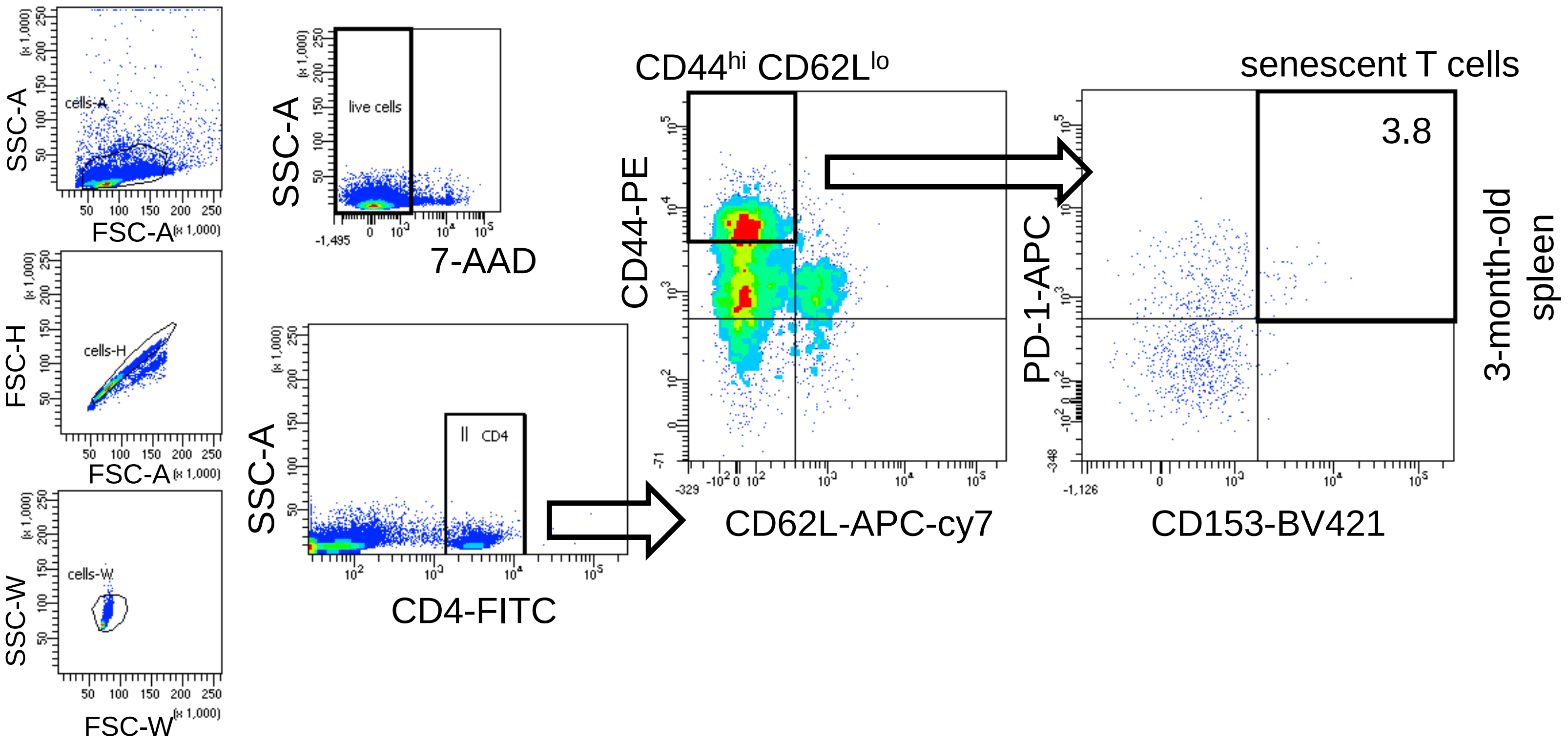

Mice were fed a normal diet. Senescent T cells (referred to as SA-T cells) were defined as PD-1^{+} CD153^{+} cells in CD4^{+}, CD44^{hi}, CD62L^{lo} cells. For gating of senescent T cells, data were generally gated in a linear fashion as follows: 1) FSC, SSC and 7-AAD were chosen to exclude debris, cell-doublets and dead cells. 2) CD4 x SSC to include CD4^{+} events, 3) CD62L x CD44 to include CD44(high) CD62L(low) events, 4) CD153 x PD-1 to include CD153^{+} PD-1^{+} events.

Supplementary Figure 3

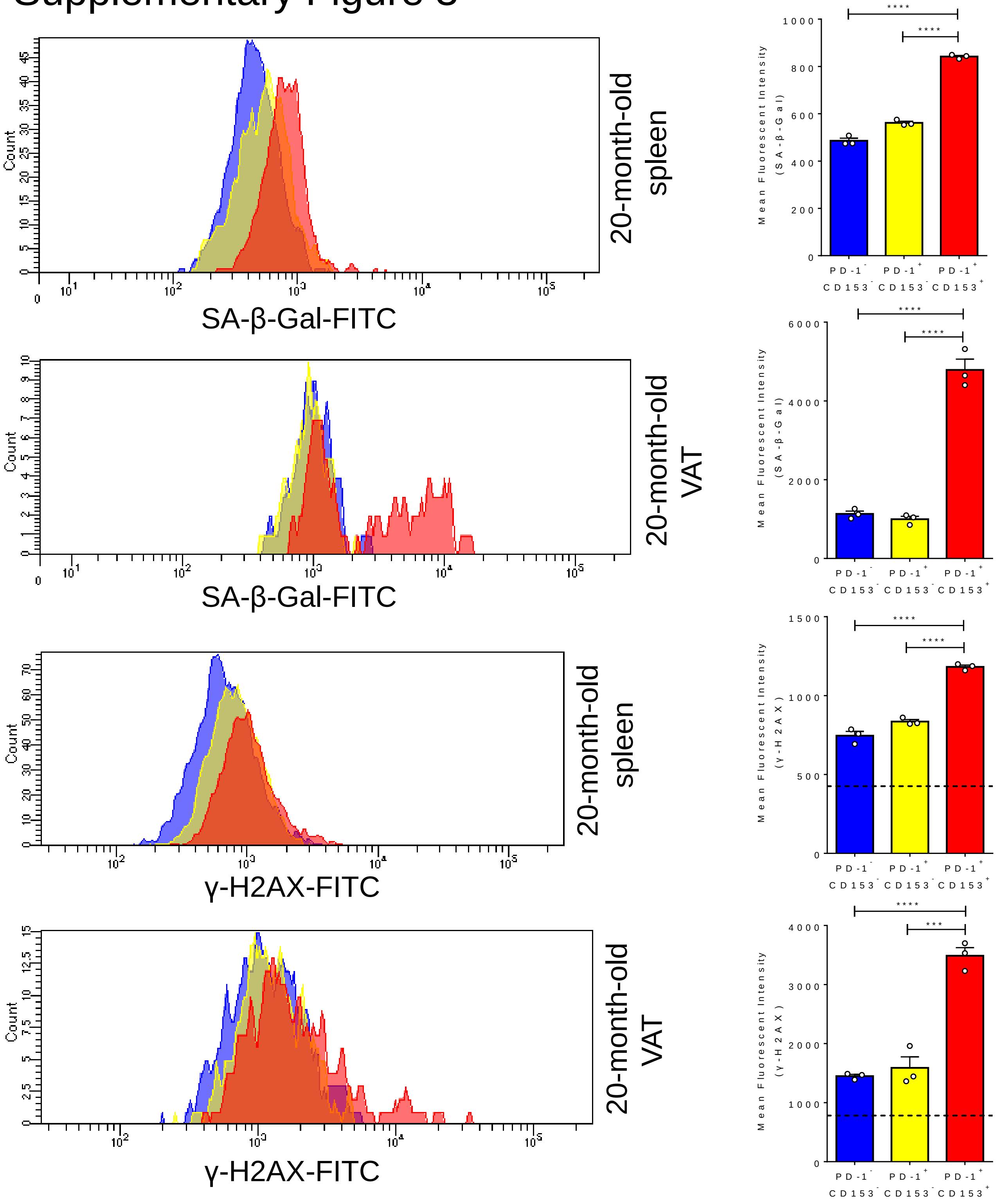

Analysis of SA-β-gal activity and γ-H2AX expression on CD4^{+} CD44^{hi} cells. Data was obtained from splenic tissues and VAT of C57BL/6J male mice (n = 3) at the age of 20 months fed with a normal diet. CD4^{+} CD44^{hi} cells were classified as follows: PD-1^{-} CD153^{-} cells (blue), PD-1^{+} CD153^{-} cells (yellow) and PD-1^{+} CD153^{+} cells (red, senescent T cells). All the data are expressed as the mean ± SEM. Statistical evaluation was performed by two-sided; Tukey's multiple comparison test; ***$p < 0.001$; ****$p < 0.0001$; dash line, the mean fluorescent intensity of IgG isotype control.

Supplementary Figure 4

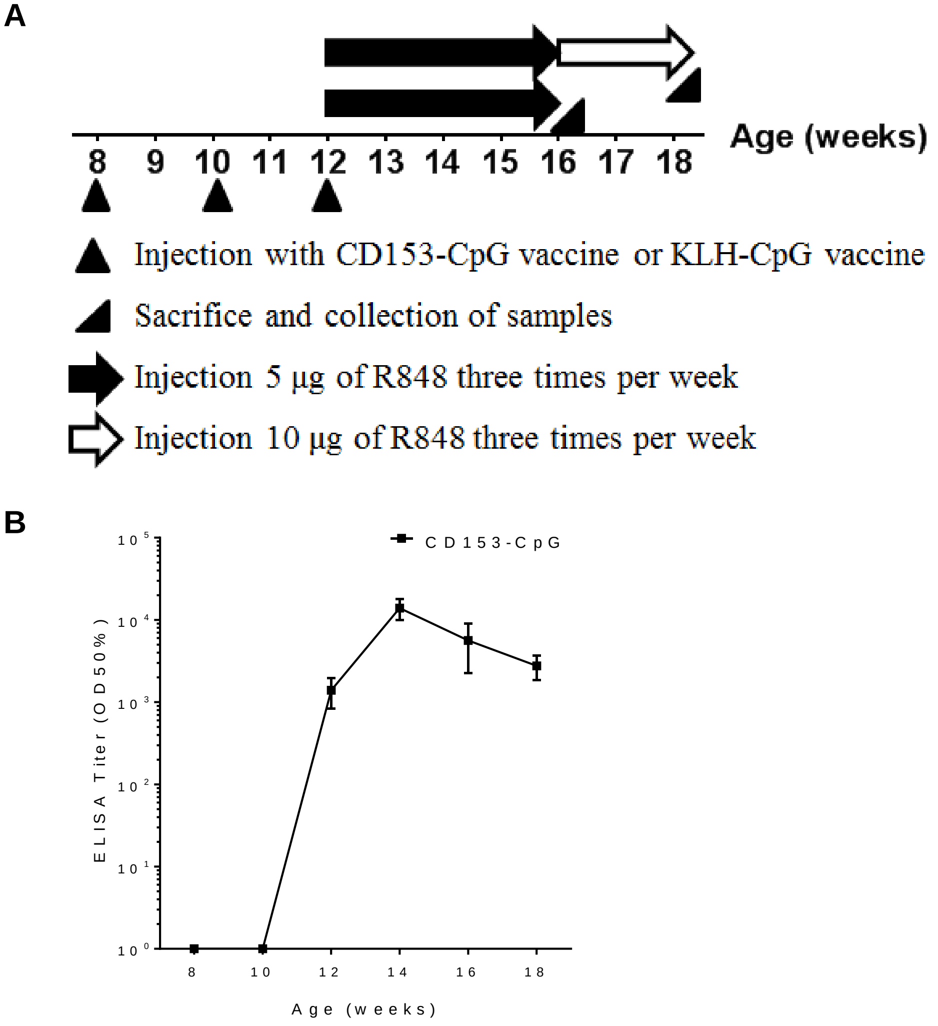

(**A**) Time course of R848 (TLR7 ligand) administration and injection of vaccines. Twelve-week-old male C57BL/6J mice and female C57BL/6N mice were intraperitoneally administered R848 three times per week. Mice sacrificed at the age of 16 weeks were administered 5 µg of R848 for 4 weeks, and mice sacrificed at the age of 18 weeks were administered 10 µg of R848 for an additional 2 weeks. All immunized mice were vaccinated with the CD153-CpG vaccine or the KLH-CpG vaccine at the ages of 8, 10, and 12 weeks. The PBS control group was unvaccinated and administered PBS instead of R848. The R848 control group was unvaccinated, while the vaccinated group was administered R848. (**B**) The titer against CD153-BSA in female C57BL/6N mice immunized with the CD153-CpG vaccine (n = 6, at the ages of 8, 10, 12, and 14 weeks; n = 3, at the ages of 16 and 18 weeks) during R848 administration. The titer is expressed as the dilution of serum providing half-maximal binding (OD 50%). All the data are expressed as the mean ± SEM.

Supplementary Figure 5

A

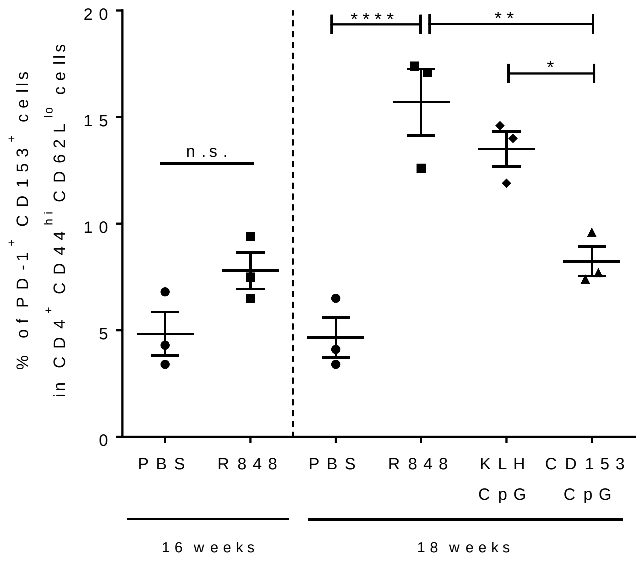

B

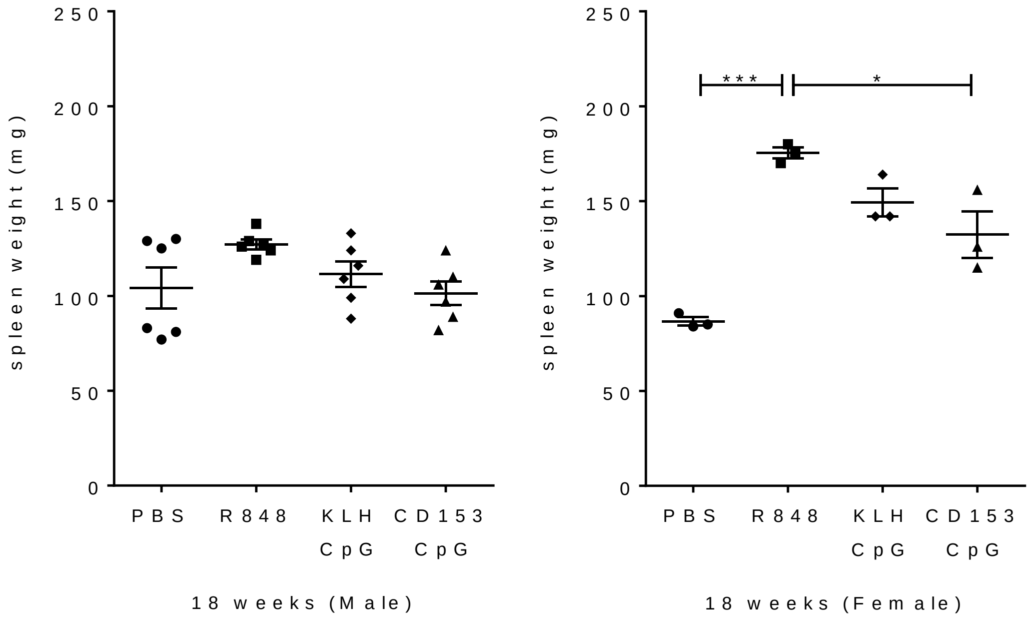

(**A**) The proportion of senescent T cells induced by R848 administration in splenic tissues of female C57BL/6N mice (n = 3 in each group) at the ages of 18 weeks with or without the CD153-CpG vaccine or the KLH-CpG vaccine. 16 weeks; PBS vs. R848, p = 0.089. 18 weeks; PBS vs. R848, p = 0.00030; R848 vs. CD153-CpG, p = 0.0045; KLH-CpG vs. CD153-CpG, p = 0.031.(**B**) The spleen weights of male C57BL/6J mice (n = 6 in each group) and female C57BL/6N mice (n =3 in each group) administered R848 at the age of 18 weeks with or without the CD153-CpG vaccine or the KLH-CpG vaccine. 18weeks (Female); PBS vs. R848, p = 0.00010; R848 vs. CD153-CpG, p = 0.014. All the data are expressed as the mean ± SEM. Statistical evaluation was performed by two-sided; unpaired two-tailed t-test (**A** in 16 weeks); Tukey's multiple comparison test (**A** in 18 weeks and **B**); *p < 0.05; **p < 0.01; ***p < 0.001; ****p < 0.0001; n.s., not significant.

Supplementary Figure 6

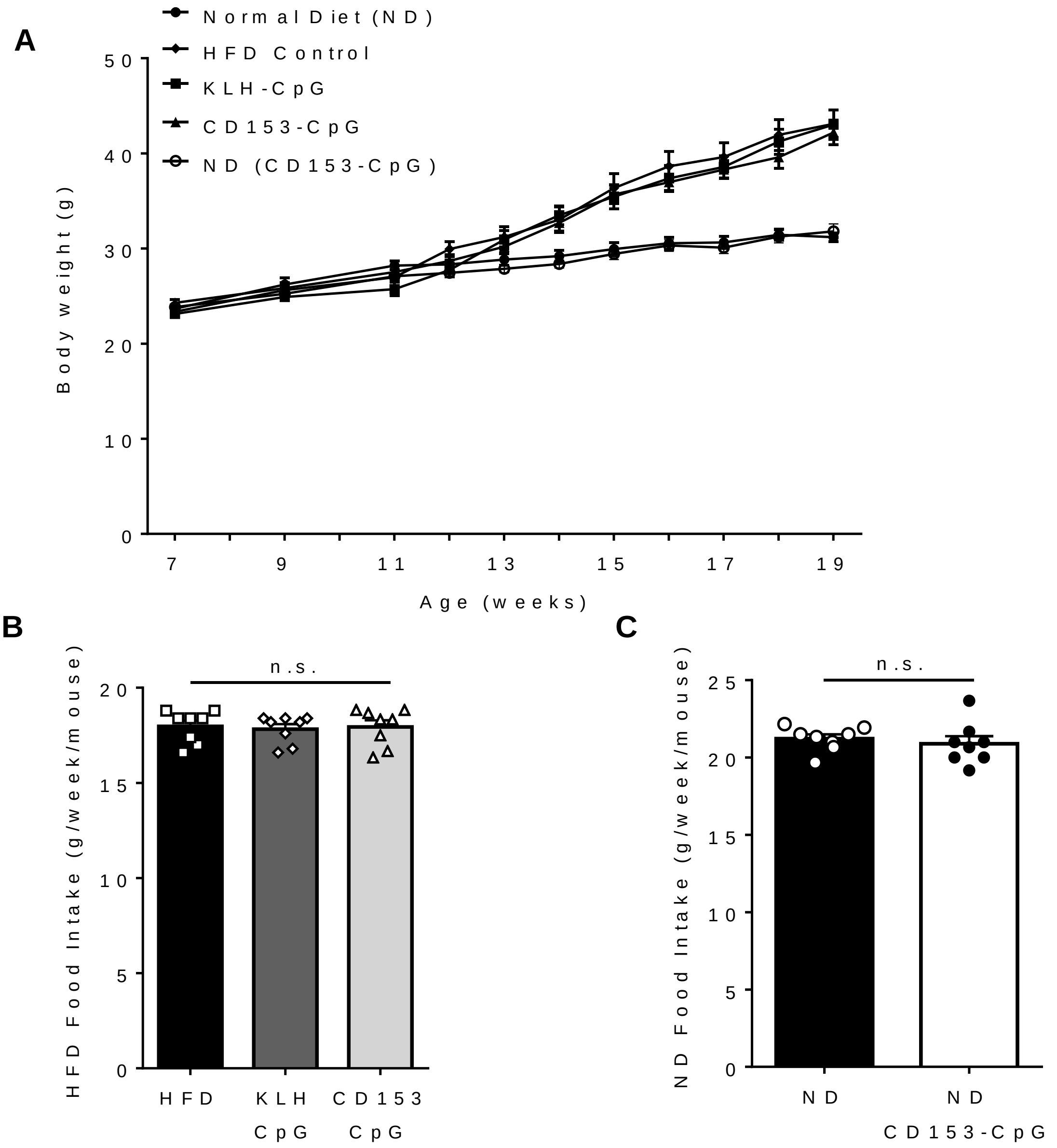

(**A**) The body weights of mice in the ND control group (n = 6), mice in the ND (CD153-CpG) group (n = 6), mice in the HFD control group (n = 5) and mice immunized with the CD153-CpG vaccine (n = 6) or the KLH-CpG vaccine (n = 5) before metabolic measurement under pair-feeding condition. All mice except for those in the ND and ND (CD153-CpG) group were fed a HFD from the age of 11 weeks. **(B)** The weekly average amount of HFD intake per mouse (g/week/mouse) in mice immunized with the CD153-CpG vaccine or the KLH-CpG vaccine during HFD loading under pair-feeding condition. Data were collected from 11 to 19 weeks of age (n = 8 in each group). (**C**) The weekly average amount of ND intake per mouse (g/week/mouse) in mice between immunized with or without the CD153-CpG vaccine. Data were collected from 11 to 19 weeks of age (n = 8 in each group). All the data are expressed as the mean ± SEM. Statistical evaluation was performed by two-sided; unpaired two-tailed t-test (**C**); Tukey's multiple comparison test (**A** and **B**); n.s., not significant.

Supplementary Figure 7

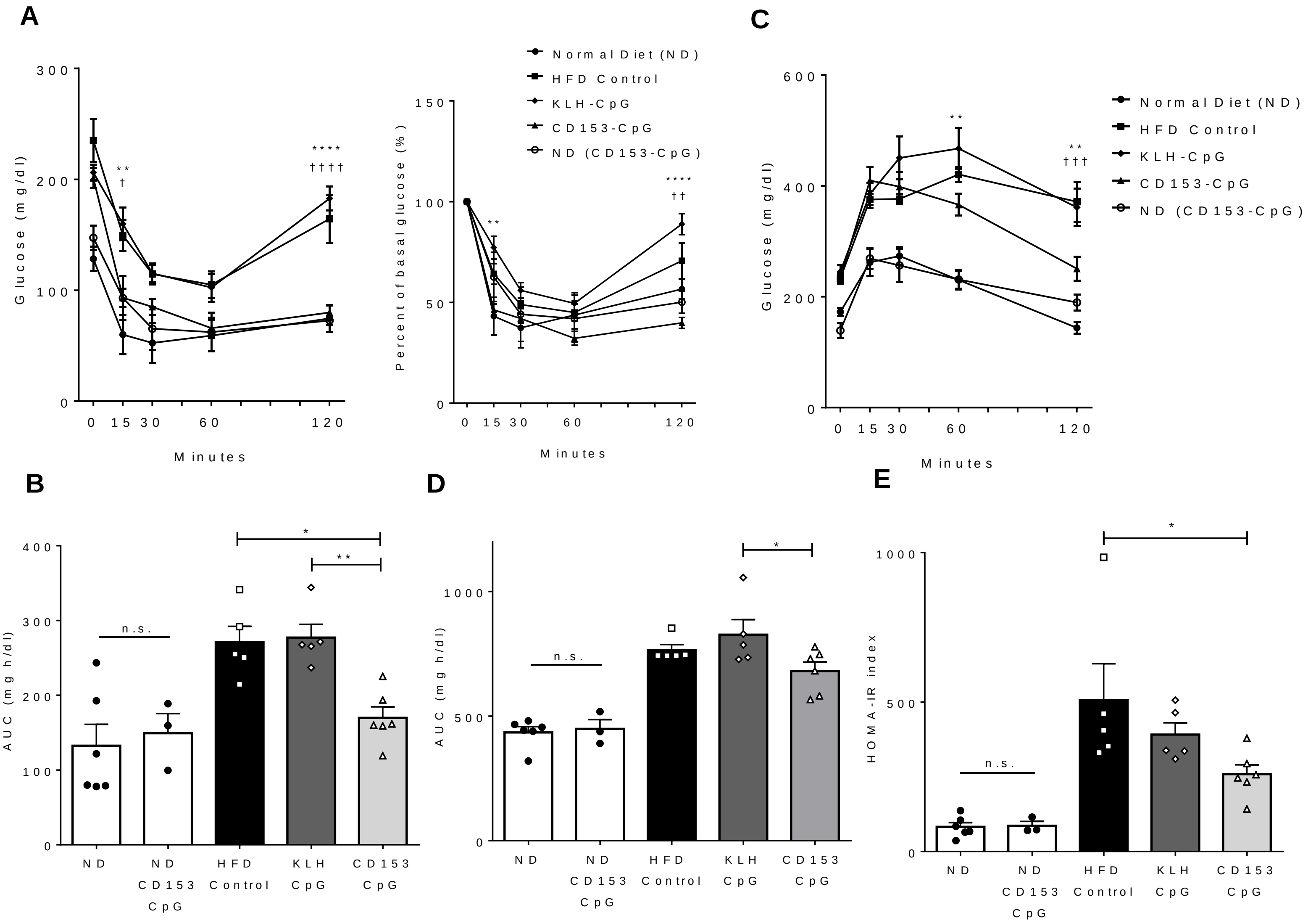

(**A–E**) Evaluation of glucose tolerance and insulin sensitivity in HFD-loaded mice immunized with the CD153-CpG vaccine under pair-feeding condition. ND control group, n = 6; ND (CD153-CpG) group, n = 6; HFD control group, n = 5; CD153-CpG vaccine group, n = 6; KLH-CpG vaccine group, n = 5. (**A**) Blood glucose concentrations in mice immunized with the CpG-conjugated vaccine as determined by the intraperitoneal insulin tolerance test (ipITT). *; the CD153-CpG group vs. the KLH-CpG group. †; the CD153-CpG group vs. the HFD control group. **(B)** Area under the curve (AUC) of blood glucose levels as determined by the ITT. (**C**) Blood glucose concentrations in mice immunized with the CpG-conjugated vaccine as determined by the oral glucose tolerance test (OGTT). *; the CD153-CpG group vs. the KLH-CpG group. †; the CD153-CpG group vs. the HFD control group. (**D**) AUC of blood glucose levels as determined by the OGTT. (**E**) HOMA-IR index after 6 hours of fasting. The AUC was estimated using the trapezoidal rule. All the data are expressed as the mean ± SEM. Statistical evaluation was performed by two-sided; Bonferroni correction, ND vs. ND-CD153-CpG, HFD control vs. KLH-CpG vs. CD153-CpG (**B**, **D** and **E**); Tukey's multiple comparison test (**A** and **C**). *p < 0.05; **p < 0.01; ****p < 0.0001; †p < 0.05; ††p < 0.01; †††p < 0.001; ††††p < 0.0001; n.s., not significant.

Supplementary Figure 8

A

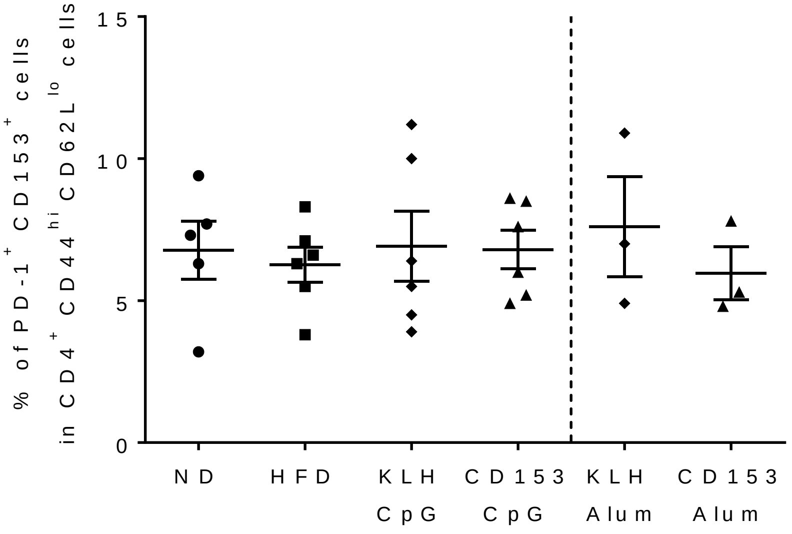

B

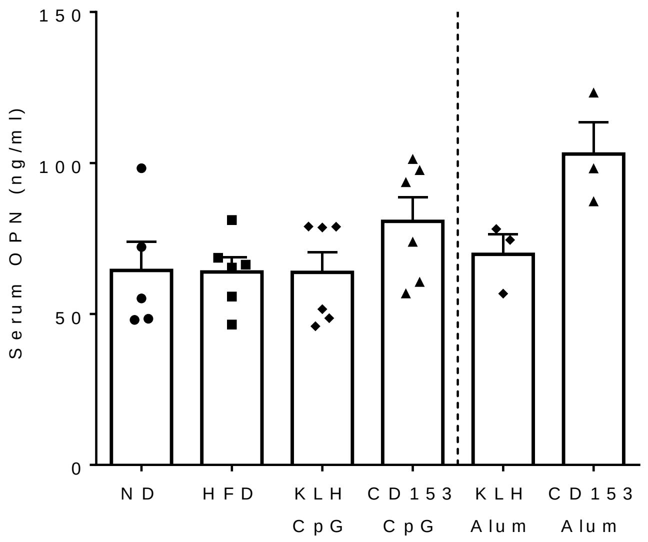

(**A**) The proportions of senescent T cells induced by HFD loading in splenic tissues of mice immunized with the Alum-conjugated vaccine or the CpG-conjugated vaccine. Senescent T cells were defined as PD-1^+ $CD153^+$ cells in $CD4^+$, $CD44^{hi}$, $CD62L^{lo}$ cells. Alum-conjugated vaccine group, n = 3 in each group; CpG-conjugated vaccine group, n = 6 in each group; ND control group, n = 5; HFD control group, n = 6. (B) The serum concentration of OPN in mice immunized with the Alum-conjugated vaccine or the CpG-conjugated vaccine. Alum-conjugated vaccine group, n = 3 in each group; CpG-conjugated vaccine group, n = 6 in each group; ND control group, n = 5; HFD control group, n = 6. All the data are expressed as the mean ± SEM. Statistical evaluation was performed by two-sided; unpaired two-tailed t-test, KLH-Alum vs. CD153-Alum (**A** and **B**); Bonferroni correction, ND vs. HFD, HFD vs. KLH-CpG, HFD vs. CD153-CpG and KLH-CpG vs. CD153-CpG (**A** and **B**).

Supplementary Figure 9

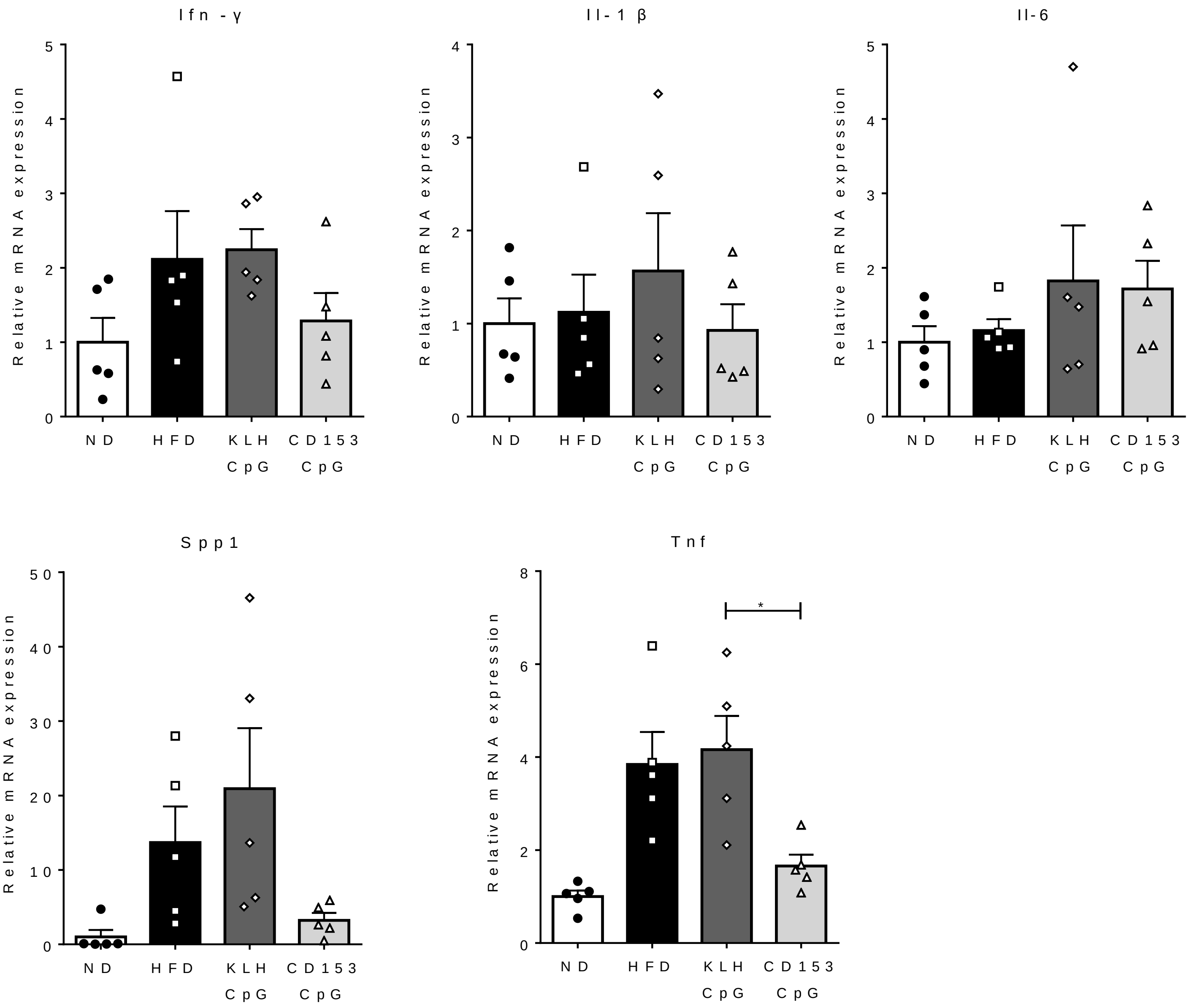

Relative *Ifn-γ*, *Il-1β*, *Il-6*, *Spp1* and *Tnf* mRNA expression levels of VAT tissues collected from the four groups of mice (ND (normal diet) control group, HFD (high-fat diet) control group, KLH-CpG vaccine group, and CD153-CpG vaccine group; n = 5 in each group) according to the time course of HFD loading (for 8 weeks) and injection of vaccines. The average mRNA expression level in ND was arbitrarily set to 1. *Tnf* ; KLH-CpG vs. CD153-CpG, $p = 0.034$. All the data are expressed as the mean ± SEM. Statistical evaluation was performed by two-sided; Bonferroni correction, HFD vs. KLH-CpG, HFD vs. CD153-CpG and KLH-CpG vs. CD153-CpG, $^*p < 0.05$.

Supplementary Figure 10

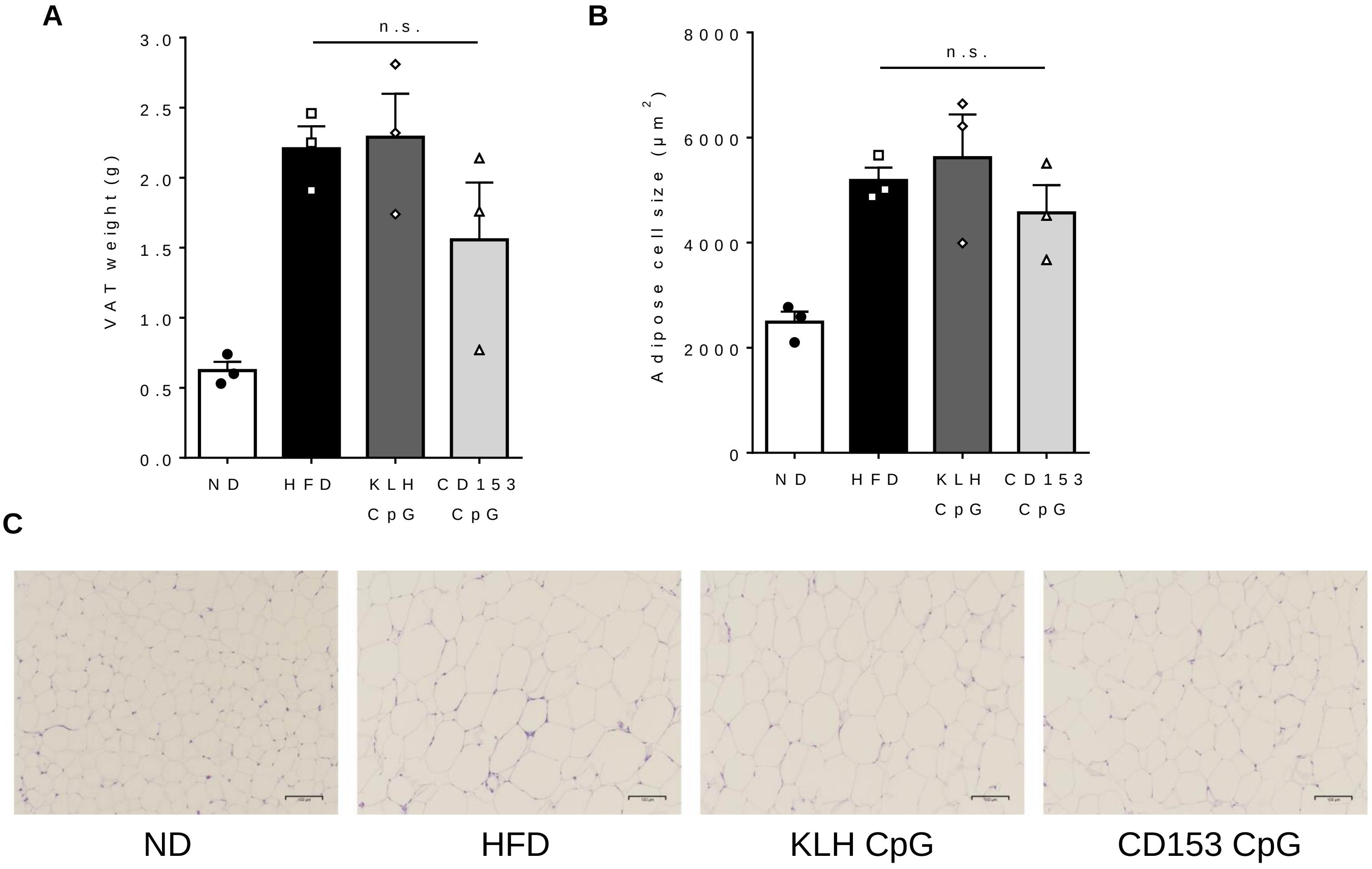

VAT were collected from the four groups of mice (ND (normal diet) control group, HFD (high-fat diet) control group, KLH-CpG vaccine group, and CD153-CpG vaccine group; n = 3 in each group) according to the time course of HFD loading and injection of vaccines. (**A**) The VAT weights among the four groups. (**B** and **C**) The average adipocyte surface areas and photomicrographs of adipose depots stained with hematoxylin and eosin (HE) among the four groups. Scale bars: 100 μm. All the data are expressed as the mean ± SEM. Statistical evaluation was performed by two-sided; Bonferroni correction, HFD vs. KLH-CpG, HFD vs. CD153-CpG and KLH-CpG vs. CD153-CpG (**A** and **B**); n.s., not significant.

Supplementary Figure 11

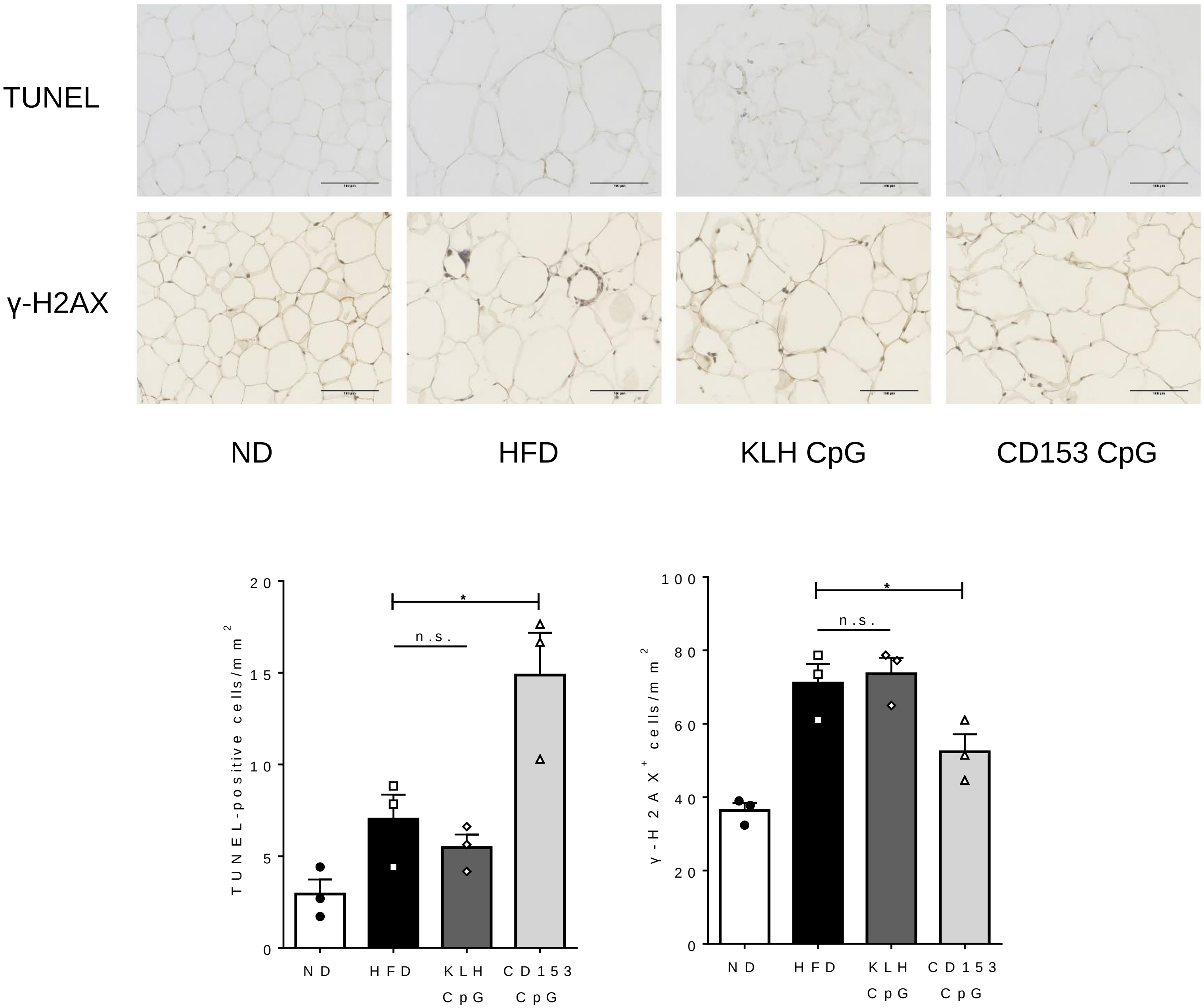

VAT were collected from the four groups of mice (ND (normal diet) control group, HFD (high-fat diet) control group, KLH-CpG vaccine group, and CD153-CpG vaccine group; n = 3 in each group) according to the time course of HFD loading and injection of vaccines. Quantification of TUNEL and γ-H2AX positive cells in the VAT among the four groups. 30 fields per sample. TUNEL and γ-H2AX (brown); scale bars: 100 µm. TUNEL; HFD vs. KLH-CpG, $p > 0.99$; HFD vs. CD153-CpG, $p = 0.014$; KLH-CpG vs. CD153-CpG, $p = 0.0051$. γ-H2AX; HFD vs. KLH-CpG, $p > 0.99$; HFD vs. CD153-CpG, $p = 0.045$; KLH-CpG vs. CD153-CpG, $p = 0.024$. All the data are expressed as the mean ± SEM. Statistical evaluation was performed by two-sided; Bonferroni correction, HFD vs. KLH-CpG, HFD vs. CD153-CpG and KLH-CpG vs. CD153-CpG, *$p < 0.05$; n.s., not significant.

Supplementary Figure 12

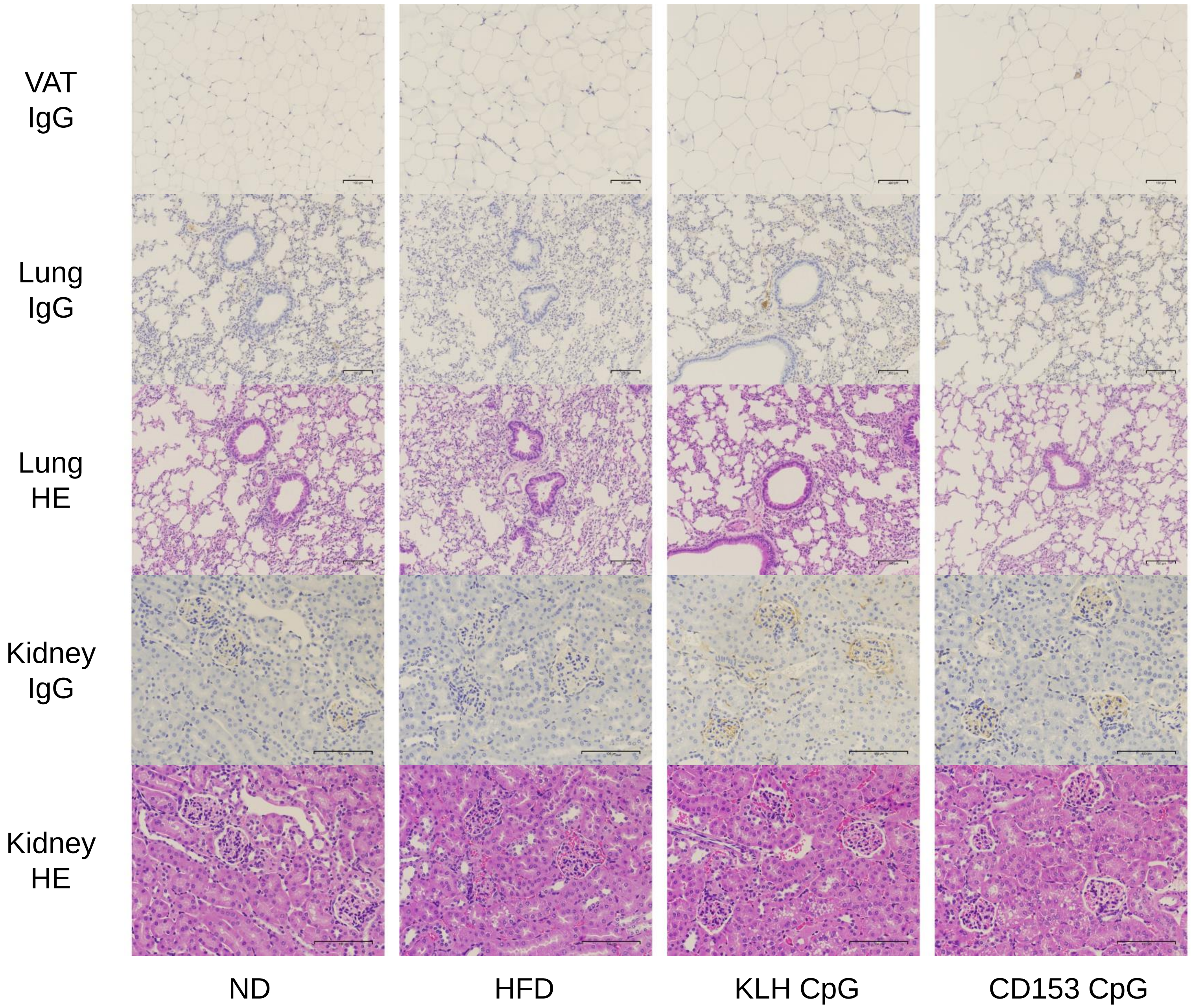

VAT, kidney tissues and lung tissues were collected from the four groups of mice (ND (normal diet) control group, HFD (high-fat diet) control group, KLH-CpG vaccine group, and CD153-CpG vaccine group; n = 3 in each group) according to the time course of HFD loading and injection of vaccines. Photomicrographs of adipose depots stained with IgG, kidney tissues stained with HE or IgG and lung tissues stained with HE or IgG among the four groups are shown. IgG (brown); scale bars: 100 µm.

Supplementary Figure 13

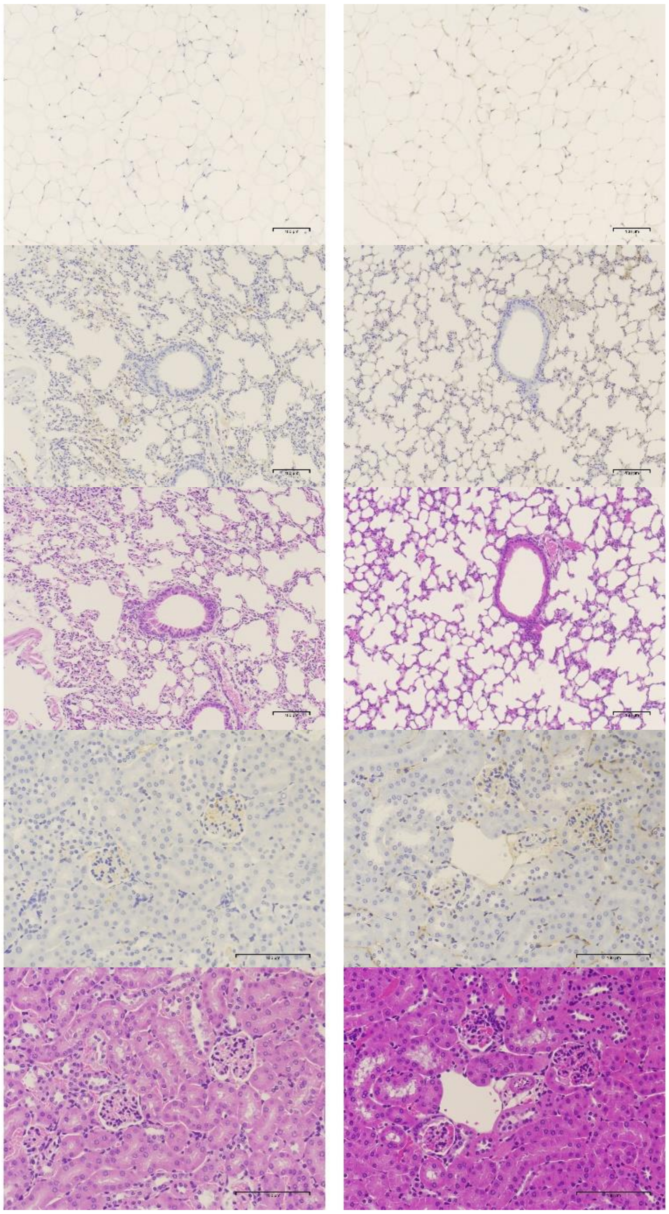

VAT, kidney tissues and lung tissues were collected from the two groups of mice (ND (normal diet) control group, ND with CD153-CpG vaccine group; n = 3 in each group) according to the time course of HFD loading and injection of vaccines. Photomicrographs of adipose depots stained with IgG, kidney tissues stained with HE or IgG and lung tissues stained with HE or IgG between the two groups are shown. IgG (brown); scale bars: 100 µm.

Supplementary Figure 14

A

CD153#D peptide amino acid sequence

Mouse VVQKKDSTPN ^{76} TTEKAPLKGG ^{85} NCSEDLFCT

Human VVQRTDSIPN ^{71} SPDNVPLKGG ^{80} NCSEDLLCI

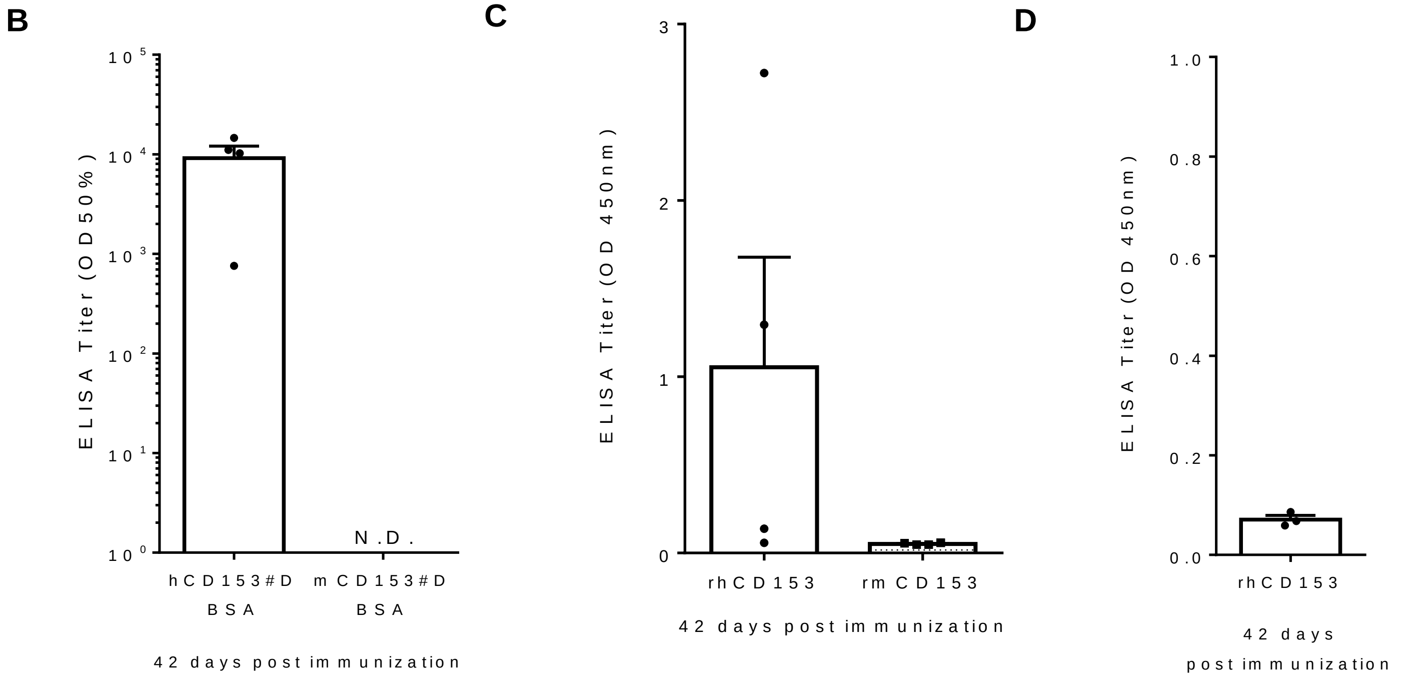

Seven-week-old C57BL/6J mice were immunized with the mouse or human CD153#D-Alum vaccine on days 0, 14 and 28. The mouse or human CD153-Alum vaccine was composed of 30 μg of the CD153 peptide conjugated to KLH (200–300 μg) and mixed with Alum adjuvant. (**A**) Amino acid sequence of mouse CD153#D peptide (76–85 aa) and human CD153#D peptide (71–80 aa). (**B**) The titer of human CD153 antibody against human or mouse CD153-BSA is expressed as the dilution of serum providing half-maximal binding (OD 50%) on days 42 postimmunization (n = 4 in each sample). (**C**) The titer of human CD153 antibody against recombinant mouse (rm-) or human (rh-) CD153 is expressed as the OD at 450 nm on days 42 postimmunization (n = 4 in each sample). (**D**) The titer of mouse CD153 antibody against rhCD153 is expressed as the OD at 450 nm on days 42 postimmunization (n = 3). All the data are expressed as the mean ± SEM; N.D., not detected.

Supplementary Figure 15. Uncropped Original Scans

Figure 1B

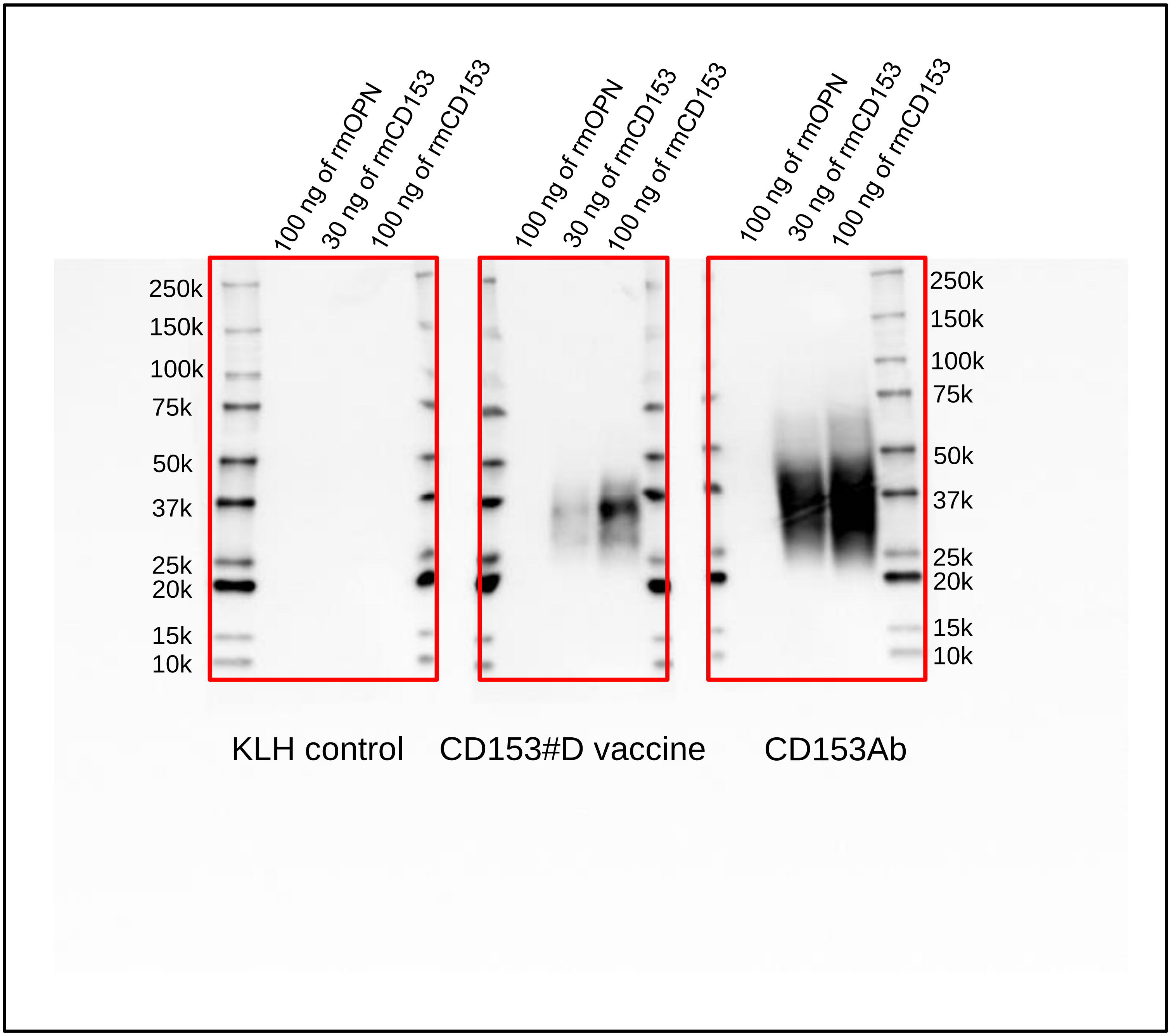

Supplementary Table 1. TaqMan primers

Gene Symbol(s)	AssayID	Gene Name(s)	Gene Alias(es)
GAPDH	Mm99999915_g1	glyceraldehyde-3-phosphate dehydrogenase	GAPD
IFNG	Mm01168134_m1	interferon gamma	IFN-G, IFG
IL1B	Mm99999061_mH	interleukin 1 beta	IL-1beta, IL-1B
IL6	Mm00446190_m1	interleukin 6	IL-6
SPP1	Mm00436767_m1	secreted phosphoprotein 1	2AR, APL-1, BNSP, BSPI, BSP, ETA-1, ETA, OP, OPN, OPNL, RIC, SPP-1
TNF	Mm99999068_m1	tumor necrosis factor	DIF, TNF-A, TNF-alpha, TNFSF2, TNFalpha, TNFA, TNFSF1A
