# Supplementary material for: The CD153 vaccine is a senotherapeutic option for preventing the accumulation of senescent T cells in mice
Source: Nat Commun. 2020 May 18;11:2482. doi: 10.1038/s41467-020-16347-w (PMC7235045; doi:10.1038/s41467-020-16347-w)
Supplement: Supplementary file 3 — Reporting Summary [file 41467_2020_16347_MOESM3_ESM.pdf]

nature research

Corresponding author(s): Hironori Nakagami

Last updated by author(s): Mar 14, 2020

Reporting Summary

Nature Research wishes to improve the reproducibility of the work that we publish. This form provides structure for consistency and transparency in reporting. For further information on Nature Research policies, see Authors & Referees and the Editorial Policy Checklist .

Statistics

For all statistical analyses, confirm that the following items are present in the figure legend, table legend, main text, or Methods section.

n/a	Confirmed	
☐	☒	The exact sample size (*n*) for each experimental group/condition, given as a discrete number and unit of measurement
☐	☒	A statement on whether measurements were taken from distinct samples or whether the same sample was measured repeatedly
☐	☒	The statistical test(s) used AND whether they are one- or two-sided<br>*Only common tests should be described solely by name; describe more complex techniques in the Methods section.*
☒	☐	A description of all covariates tested
☐	☒	A description of any assumptions or corrections, such as tests of normality and adjustment for multiple comparisons
☐	☒	A full description of the statistical parameters including central tendency (e.g. means) or other basic estimates (e.g. regression coefficient) AND variation (e.g. standard deviation) or associated estimates of uncertainty (e.g. confidence intervals)
☐	☒	For null hypothesis testing, the test statistic (e.g. *F*, *t*, *r*) with confidence intervals, effect sizes, degrees of freedom and *P* value noted<br>*Give P values as exact values whenever suitable.*
☒	☐	For Bayesian analysis, information on the choice of priors and Markov chain Monte Carlo settings
☒	☐	For hierarchical and complex designs, identification of the appropriate level for tests and full reporting of outcomes
☒	☐	Estimates of effect sizes (e.g. Cohen's *d*, Pearson's *r*), indicating how they were calculated

Our web collection on statistics for biologists contains articles on many of the points above.

Software and code

Policy information about availability of computer code

Data collection	ELISA: iMark microplate absorbance reader (Bio-Rad), Western blot: LAS 1000 instrument (Fuji Film), Oxygen consumption: MK-5000RQ (Muromachi Kikai), Flow cytometry: BD FACS Canto II (BD Biosciences), Realtime PCR: 7900HT Fast Real-Time PCR System (Applied Biosystems), Imaging: FSX100 (Olympus)
Data analysis	ELISA: MPM6 version 6.1 (Bio-Rad), Western blot: Multi Gauge software version 3.2 (Fuji Film), Oxygen consumption: MMS-2 software (Muromachi Kikai), Flow cytometry: BD FACS Diva software version 8.0.1 (BD Biosciences), Realtime PCR: SDS software version 2.4 (Applied Biosystems), Imaging: FSX-BSW version 3.2.0.0 (Olympus),Curve fitting: ImageJ version 1.48, Area measurement in immunohistochemical images: ImageJ version 1.48, Statistical analyses: Prism GraphPad version 6.07 (GraphPad Software).

For manuscripts utilizing custom algorithms or software that are central to the research but not yet described in published literature, software must be made available to editors/reviewers. We strongly encourage code deposition in a community repository (e.g. GitHub). See the Nature Research guidelines for submitting code & software for further information.

Data

Policy information about availability of data

All manuscripts must include a data availability statement. This statement should provide the following information, where applicable:

- Accession codes, unique identifiers, or web links for publicly available datasets
- A list of figures that have associated raw data
- A description of any restrictions on data availability

All data suppoeting the findings in this study are available from the corresponding author upon reasonable request. The source data underlying all Figures and Supplement Figures are provided in a Source Data File.

Field-specific reporting

Please select the one below that is the best fit for your research. If you are not sure, read the appropriate sections before making your selection.

- [x] Life sciences
- [ ] Behavioural & social sciences
- [ ] Ecological, evolutionary & environmental sciences

For a reference copy of the document with all sections, see nature.com/documents/nr-reporting-summary-flat.pdf

Life sciences study design

All studies must disclose on these points even when the disclosure is negative.

Sample size

No statistical method was used to determine the sample size. Sample size for each experiment was determined based on the previous reports.

For the experiment of TLR7 ligand administration (Fig. 1E and Supplementary Fig. 4 and 5), including vaccination, ELISA, flow cytometry analysis and spleen-weight measurement, we determined the sample size based on the previous similar study (Sakamoto, K. et al. J. Immunol. 2016).

For the experiment of high fat-induced diabetes model, including vaccination (Fig. 2, 3 and 4A and Supplementary Fig. 6, 7, 8 and 9), ELISA, flow cytometry analysis, real-time quantitative PCR, evaluation of glucose metabolism and oxygen consumption, we determined the sample size based on the previous similar study (Shirakawa, K. et al. J. Clin. Invest. 2016).

For the screening of peptide vaccines for mouse or human CD153 (Fig. 1A and 1C and Supplementary Fig. 1B, 14B, 14C and 14D), we determined the sample size (N = 3–6) based on our previous studies on vaccine (e.g. Koriyama, H. et al. Hypertension 2015; Pang, Z. et al. Proc. Natl. Acad. Sci. U.S.A. 2014; Nakagami, F. et al. PLoS One 2013).

For the evaluation of senescent cells (Fig. 1D and Supplementary Fig. 3), we determined the sample size based on the previous similar study (Shirakawa, K. et al. J. Clin. Invest. 2016).

For the immunohistochemical staining (Fig. 4C and 4D and Supplementary Fig. 10, 11, 12 and 13), we determined the sample size (N =3) based on the previous similar study (Shirakawa, K. et al. Pros One 2017) and cost limitations.

For the CDC assay (Fig.4B), we determined the sample size (N =3) based on the previous studies dealing with CDC assay (e.g. van Meerten, T. et al. Clin. Cancer Res. 2006).

Data exclusions

Realtime PCR: On analysis of IL-1 beta, IL-6 and IFN-g, some outlier wells were omitted to obtain a highly accurate standard curve.
Oxygen consumption: Blank data caused by the machine specifications were excluded. The data for a few hours after starting metabolic measurement was excluded because mice just moved into the metabolic cages were restless and hyperactive. Extreme outliers, such as transient plunged (VO2 > 2.5 ml/min) or leaped (VO2 < 1.0 ml/min) oxygen consumption, caused by artifacts were also excluded.
These exclusion criteria was established prior to any data generation.

Replication

Experiments were performed and repeated by different lab members, and all findings are reproduced.
To ensure reproducibility of our data, all experiments were replicated at least twice.

Randomization

Before starting an animal experiment (screening of peptide vaccines, TLR7 ligand administration and high fat-induced diabetes model), all animal groups were made with set of animals of the same sex, age and similar body weight. All the experimental samples, such as blood and organ tissues, were collected from all mice in the randomized groups.
For the analysis of the immunohistochemical staining images, all images were randomly captured from each sample section.

Blinding

For the screening of peptide vaccines, the experiment of TLR7 ligand administration and the experiment of high fat-induced diabetes model, the investigators were not blinded during these studies because the vaccines and chemicals had to be administrated to the same mouse periodically and body weight measurements were taken at the same time.
For sample analysis using analysis equipments (e.g. ELISA, flow cytometry), there was no blinding because the investigators performing the analysis were aware of which experimental group each sample was collected from. However, there is limited potential for the investigators to affect the data when the same experimental procedure and computational approach was applied for all samples.
For the analysis of the immunohistochemical staining images, the image files were renamed and assigned a random numerical code so that the investigators were blinded during all image analysis.

Reporting for specific materials, systems and methods

We require information from authors about some types of materials, experimental systems and methods used in many studies. Here, indicate whether each material, system or method listed is relevant to your study. If you are not sure if a list item applies to your research, read the appropriate section before selecting a response.

Materials & experimental systems

n/a	Involved in the study	
	✗	Antibodies
	✗	Eukaryotic cell lines
✗		Palaeontology
	✗	Animals and other organisms
✗		Human research participants
✗		Clinical data

Methods

n/a	Involved in the study	
✗		ChIP-seq
	✗	Flow cytometry
✗		MRI-based neuroimaging

Antibodies

Antibodies used

ELISA: horseradish peroxidase (HRP)-conjugated antibodies specific for mouse IgG (1:1000; GE Healthcare, UK) and anti-mouse IgG subclass-specific HRP-conjugated antibodies (1:1000; IgG1, IgG2b, IgG2c and IgG3, Abcam).

Western blot: anti-mouse CD153 antibody (0.05 μg/ml, Cat# AF732; R&D Systems) and horseradish peroxidase (HRP)-conjugated antibodies specific for mouse IgG (1:2000; GE Healthcare, UK).

CDC assay: anti-mouse CD153 antibody (functional grade, RM153, Cat# 14-1531-85; eBioscience) and anti-mouse major histocompatibility complex (MHC) class I antibody (H-2Kd/H-2Dd, functional grade, 34-1-2S, Cat# 16-5998-82 Lot# 2003128; eBioscience).

Flow cytometry: anti-mouse CD16/CD32 antibody (purified, 2.4G2, Cat# 553142, Lot# 5154726; BD Biosciences), anti-mouse CD4-FITC antibody (RM4-4, Cat# 553055; BD Biosciences), anti-mouse CD44-PE antibody (IM7, Cat# 553134; BD Biosciences), anti-mouse CD153-BV421 antibody (RM153, Cat# 740059; BD Biosciences), anti-mouse CD62L-APC-Cy7 antibody (MEL-14, Cat# 104428; Bio Legend), anti-mouse PD-1-APC antibody (29F.1A12, Cat# 135210; Bio Legend), anti-mouse CD4-APC-Cy7 antibody (RM4-5, Cat# 565650; BD Biosciences) and anti-γ-H2AX-Alexa Fluor 488 antibody (2F3, Cat# 613405; Bio Legend).

Immunohistochemistry: anti-F4/80 antibody (CI:A3-1, Cat# ab6640; Abcam), anti-CD153 antibody (Cat# orb156296; Biorbyt), anti-γ-H2AX antibody (Cat# ab2893; Abcam).

Validation

For our anti-mouse CD153 antibody from CD153 peptide vaccine, the validation was performed by ELISA and western blot analysis. All commercially available antibodies used are validated.

ELISA
horseradish peroxidase (HRP)-conjugated antibodies specific for mouse IgG (GE Healthcare): suitable for protein blotting, ELISA and immunocytochemistry. Manufacturer website provides the datasheet "NA931V, Amersham ECL Anti-Mouse IgG, Horseradish Peroxidase-Linked Species-Specific Whole Antibody (from sheep)". The datasheet states this antibody was used in 4 citations.
anti-mouse IgG subclass-specific HRP-conjugated antibodies (Abcam): tested for immunocytochemistry, immunohistochemistry (paraffin), ELISA and western blotting. These antibodies were used in more than 10 citations (IgG1, ab97240), 9 citations (IgG2b ,ab97250), 6 citations (IgG2c ,ab97255) and 6 citations (IgG3 ,ab97260).

Western blot
anti-mouse CD153 antibody (R&D Systems): suitable for western blotting and neutralization assay. This antibody was used 3 citations.
horseradish peroxidase (HRP)-conjugated antibodies specific for mouse IgG (GE Healthcare): suitable for protein blotting, ELISA and immunocytochemistry. Manufacturer website provides the datasheet "NA931V, Amersham ECL Anti-Mouse IgG, Horseradish Peroxidase-Linked Species-Specific Whole Antibody (from sheep)". The datasheet states this antibody was used in 4 citations.

CDC assay
anti-mouse CD153 antibody (eBioscience): reported for use in flow cytometry, immunohistochemistry (frozen), Immunoprecipitation, neutralization and functional assay. This antibody was used in one citation.
anti-mouse MHC class I antibody (eBioscience): suitable for flow cytometry and functional assay. This antibody was used in 3 citations.

Flow cytometry
anti-mouse CD16/CD32 antibody (BD Biosciences): suitable for flow cytometry, routinely tested for blocking, tested for immunohistochemistry (frozen), and reported for immunoprecipitation. This antibody was used in more than 10 citations.
anti-mouse CD4 antibody (BD Biosciences): routinely tested for flow cytometry. This antibody was used in more than 10 citations.
anti-mouse CD44 antibody (BD Biosciences): routinely tested for flow cytometry. This antibody was used in more than 10 citations.
anti-mouse CD153 antibody (BD Biosciences): qualified for flow cytometry. This antibody was used in 3 citations.
anti-mouse CD62L antibody (Bio Legend): quality tested for flow cytometry. This antibody was used in more than 10 citations.
anti-mouse PD-1 antibody (Bio Legend): quality tested for flow cytometry. This antibody was used in more than 10 citations.
anti-γ-H2AX antibody (Bio Legend): quality tested for intracellular staining for flow cytometry. This antibody was used in more

than 10 citations.

Immunohistochemistry
anti-F4/80 antibody (Abcam): tested for western blotting, immunoprecipitation, radioimmunoassay, immunohistochemistry (frozen, resin or PFA perfusion fixed frozen section), flow cytometry, immunocytochemistry and immunofluorescence. This antibody was used in more than 500 citations. Although manufacturer does not recommend this antibody for immunohistochemistry (paraffin), this antibody has been already used in about 50 citations.
anti-CD153 antibody (Biorbyt): tested for ELISA, immunocytochemistry, immunohistochemistry (paraffin) and immunofluorescence. This antibody was used in no citation.
anti-γ-H2AX antibody (Abcam):tested for western blotting, immunoprecipitation, immunohistochemistry (paraffin or PFA perfusion fixed frozen section), immunocytochemistry and immunofluorescence. This antibody was used in more than 200 citations.

Eukaryotic cell lines

Policy information about cell lines

Cell line source(s)	Murine macrophage cell line RAW 264.7 was obtained from the American Type Culture Collection (ATCC).
Authentication	None of the cell line used was authenticated in this study.
Mycoplasma contamination	RAW 264.7 cells were tested negative for mycoplasma contamination.
Commonly misidentified lines (See ICLAC register)	RAW 264.7 cells were not listed in ICLAC Register of Misidentified Cell Lines version 9.

Animals and other organisms

Policy information about studies involving animals; ARRIVE guidelines recommended for reporting animal research

Laboratory animals	Seven (or eight)-week-old male C57BL/6J mice and eight-week-old female C57BL/6N mice were purchased from CLEA Japan Inc. and housed in a temperature-, humidity- and light cycle-controlled facility (23°C ± 1°C; 55% ± 10 %; light, 8:00–20:00; dark, 20:00–8:00).
Wild animals	No wild animals were used in the study.
Field-collected samples	No field collected samples were used in the study.
Ethics oversight	All animal experimental procedures were reviewed and approved by the Institutional Animal Committee at the Department of Veterinary Science of Osaka University School of Medicine and performed in accordance with guidelines for animal experimentation at research institutes (Ministry of Education, Culture, Sports, Science and Technology, Japan), guidelines for animal experimentation at institutes (Ministry of Health, Labor and Welfare, Japan), and guidelines for the proper conduction of animal experiments (Science Council of Japan)

Note that full information on the approval of the study protocol must also be provided in the manuscript.

Flow Cytometry

Plots

Confirm that:

- [x] The axis labels state the marker and fluorochrome used (e.g. CD4-FITC).
- [x] The axis scales are clearly visible. Include numbers along axes only for bottom left plot of group (a 'group' is an analysis of identical markers).
- [x] All plots are contour plots with outliers or pseudocolor plots.
- [x] A numerical value for number of cells or percentage (with statistics) is provided.

Methodology

Sample preparation	Splenocytes were collected from mice spleen, then triturated gently and suspended in staining buffer.<br>Visceral adipose tissues (VAT) were collected from epidydimal fat pads, minced into fine pieces and incubated in a digestion buffer composed of Hank's Balanced Salt Solution (HBSS) in 10% FBS, 100 μg/ml DNase I and 200 U/ml collagenase type I (Worthington, Lakewood, NJ, USA) at 37°C for 1 hour while shaking. Digested tissue was centrifuged at 1000 x g for 10 minutes at 4°C and resuspended in staining buffer.<br>Red blood cells were removed from splenocytes and VAT using ACK erythrocyte-lysing buffer (Gibco, Grand Island, NY, USA). After lysing erythrocytes, the suspended cells were filtered through a 70-μm filter, centrifuged at 1000 x g for 10 minutes at 4°C and resuspended in staining buffer. After blocking Fc-receptors with an anti-mouse CD16/32 antibody (mouse Fc-receptor blocker; BD Biosciences) for 20 minutes at 4°C, cells were stained with a mixture of fluorescently labeled antibodies at 4°C for 40 minutes in the dark. The antibodies used were specific to CD4-FITC (RM4-4), CD44-PE (IM7), CD153-BV421 (RM153) (BD Biosciences), CD62L-APC-Cy7 (MEL-14) and PD-1-APC (29F.1A12) (Bio Legend). A 7-AAD viability staining solution was added to

exclude dead cells.
In addition, SA-β-gal assay and intracellular staining of γ-H2AX were also performed.
SA-β-gal assay was performed using a cellular senescence detection kit (SPiDER-βGal, Dojindo). Blocking Fc-receptors and staining cells with a mixture of fluorescently labeled antibodies, CD4-APC-Cy7 (RM4-5, BD Biosciences), CD44-PE, CD153-BV421 and PD-1-APC, was performed after SA-β-gal assay. Intracellular staining of γ-H2AX was performed using a fixation/permeabilization solution kit (BD Biosciences) according to the manufacturer's recommendations. Blocking Fc-receptors and staining cells with a mixture of fluorescently labeled antibodies was performed before intracellular staining of γ-H2AX-Alexa Fluor 488 (2F3, Bio Legend). 7-AAD staining was not performed in these assays because 7-AAD cannot be used when labeling intracellular molecules.

Instrument

BD FACS Canto II (BD Biosciences)

Software

BD FACS Diva software v8.0.1. (BD Biosciences)

Cell population abundance

For gating of SA-T cells, sorted cells were checked for purity using 7-AAD to exclude dead cells. Sorted samples generally had a purity over 90% in splenocytes and over 85% in VAT.
The abundance of cells in the post-sort fraction was dependent on cell-type and sorting condition. CD4+ cells in living cells were typically about 10-20% of the population in splenocytes and VAT. CD44(High) CD62L(low) cells in CD4+ cells were typically over 25% of the population in splenocytes and were typically over 60% of the population in VAT.

Gating strategy

For gating of SA-T cells, data were generally gated in a linear fashion as follows:
1) forward and side scatter parameters (FSC, SSC) were chosen to exclude debris and cell-doublets, 2) 7-AAD x SSC to exclude 7-AAD+ events (dead cells), 3) CD4 x SSC to include CD4+ events, 4) CD62L x CD44 to include CD44(high) CD62L(low) events, 5) CD153 x PD-1 to include CD153+ PD-1+ events. We also exemplified in Supplementary Figure 2.
CD44(high) events were set compared with the intensity of CD44-PE fluorescence in CD62L+ (high) events.

☒ Tick this box to confirm that a figure exemplifying the gating strategy is provided in the Supplementary Information.
